# The Role of the Extracellular Matrix and Tumor-Infiltrating Immune Cells in the Prognostication of High-Grade Serous Ovarian Cancer

**DOI:** 10.3390/cancers14020404

**Published:** 2022-01-14

**Authors:** Yuri Belotti, Elaine Hsuen Lim, Chwee Teck Lim

**Affiliations:** 1 Institute for Health Innovation and Technology, National University of Singapore, 14 Medical Drive, Singapore 117599, Singapore; yuri_belotti@bii.a-star.edu.sg; 2 Division of Medical Oncology, National Cancer Center Singapore, 11 Hospital Drive, Singapore 169610, Singapore; elaine.lim.hsuen@singhealth.com.sg; 3 Department of Biomedical Engineering, National University of Singapore, 4 Engineering Drive 3, Singapore 117583, Singapore; 4 Mechanobiology Institute, National University of Singapore, 5A Engineering Drive 1, Singapore 117411, Singapore

**Keywords:** extracellular matrix, ovarian cancer, high-grade serous ovarian carcinoma, bioinformatics, TCGA, prognostic biomarker, machine learning, precision medicine

## Abstract

**Simple Summary:**

Despite the improvements in the survival rates observed in various types of cancer in recent years, the mortality rates remain high in ovarian cancer. This is primarily caused by the advanced disease stage at presentation and the lack of effective screening methods. An important clinical objective is represented by the ability to perform a post-surgical risk stratification to identify better and more effective intervention strategies to minimize recurrence and maximize survival. Here, we sought to leverage the availability of publicly available ovarian cancer RNA sequencing data and the use of bioinformatics methods to identify a prognostic gene panel in non-metastatic high-grade serous ovarian carcinoma. Moreover, we found an association between mortality rates and tumor-infiltrating immune cells.

**Abstract:**

Ovarian cancer is the eighth global leading cause of cancer-related death among women. The most common form is the high-grade serous ovarian carcinoma (HGSOC). No further improvements in the 5-year overall survival have been seen over the last 40 years since the adoption of platinum- and taxane-based chemotherapy. Hence, a better understanding of the mechanisms governing this aggressive phenotype would help identify better therapeutic strategies. Recent research linked onset, progression, and response to treatment with dysregulated components of the tumor microenvironment (TME) in many types of cancer. In this study, using bioinformatic approaches, we identified a 19-gene TME-related HGSOC prognostic genetic panel (19 prognostic genes (*PLXNB2*, *HMCN2*, *NDNF*, *NTN1*, *TGFBI*, *CHAD*, *CLEC5A*, *PLXNA1*, *CST9*, *LOXL4*, *MMP17*, *PI3*, *PRSS1*, *SERPINA10*, *TLL1*, *CBLN2*, *IL26*, *NRG4,* and *WNT9A*) by assessing the RNA sequencing data of 342 tumors available in the TCGA database. Using machine learning, we found that specific patterns of infiltrating immune cells characterized each risk group. Furthermore, we demonstrated the predictive potential of our risk score across different platforms and its improved prognostic performance compared with other gene panels.

## 1. Introduction

According to the most recent estimates, around 314,000 new ovarian cancer (OC) cases have been diagnosed in 2020, with more than 200,000 victims [1]. In the high Human Development Index (HDI) countries, OC is the seventh most common cause of cancer-associated death in women [1]. Despite the improvements in the survival rates achieved in many solid tumors over the last decades, a meta-analysis by Vaughan et al. [2] highlighted that the 5-year overall survival from ovarian cancer has not improved substantially since 1980. The high mortality rates associated with OC are primarily due to the advanced disease stage at presentation and the lack of effective screening tools [3].

The high-grade serous subtype (HGSOC) is the most common form of ovarian cancer and often results in fatalities [4]. In non-metastatic cases where the primary cancer has been definitively treated (e.g., surgically resected), the ability to accurately risk-stratify disease recurrence has significant clinical utility in terms of follow-up and early intervention, thus impacting survival. To achieve this, a deeper understanding of the molecular factors that affect the progression of ovarian cancer is required, as they likely play a role in risk-stratification, as well as the development of novel therapeutics [5].

Over the last two decades, high volumes of genomic and proteomic data have been acquired, resulting in deeper insights into carcinogenesis and cancer progression, as well as contributing towards the field of molecular targeted therapy [6]. Cancer had been investigated primarily from a genetic and epigenetic standpoint, focusing on epithelial cells. However, there has been a recent shift of attention towards the tumor microenvironment (TME) [7]. Alterations in the organization and composition of the TME, especially in the extracellular matrix (ECM), have been linked with cancer growth, progression, and metastasis [7,8]. A more comprehensive understanding of the molecular changes occurring at the matrisomal level may aid the identification of novel characteristics that possess diagnostic, prognostic, and therapeutic values. The status of the TME may explain the heterogeneity in clinical outcomes, despite similar histopathological staging. Recent studies provided evidence that dysregulated matrisomal components enable prognostication and prediction of early stage non-small-cell lung (NSCLC) cancer [9,10]. The authors developed a 29-gene ECM-related prognostic and predictive indicator that allowed them to stratify patients into risk groups and link them with survival outcomes. This genomic tool was also shown to predict adjuvant chemotherapy (ACT) response in NSCLC patients, and, more recently, it has been associated with tumor progression, mutational load, tumor pathology, and survival across different malignancies in thousands of patient-derived samples [11,12]. Furthermore, this predictive indicator was used in addition to multi-region sequencing data from primary tumor tissues to address intratumoral phenotypic variation [13]. Moreover, increasing evidence highlighted the strong link between tumor-infiltrating immune cells and prognosis [14,15,16,17,18,19,20,21,22]. In a recent paper by Izar et al. [15], the authors analyzed scRNA-seq data from more than 45,000 cells and found expression of inflammatory programs in malignant cells both in cancer tissue samples and malignant abdominal fluid (ascites). This has important clinical implications, as ascites often develop in HGSOC patients and are associated with drug resistance and a poor prognosis [23].

In the present study, we applied a bioinformatics protocol to analyze publicly available RNA-seq datasets from The Cancer Genome Atlas (TCGA) in order to identify a novel prognostic matrisomal gene signature for high-grade serous ovarian carcinoma (HGSOC). Using a Cox proportional hazard model on matrisome-associated genes, we first defined a risk score, the HGSOC Tumor Matrisome Index (HGSOC-TMI) and stratified early stage cancer patients into risk groups. We then validated the risk score internally by using bootstrapping validation and externally on two independent microarrays datasets. Furthermore, we analyzed the expression of the HGSOC-TMI genes in two single-cell datasets from HGSOC cancer tissue and ascites samples, respectively. Using a machine-learning deconvolution method, we investigated the role of infiltrating immune cells in each risk group. We then sought to demonstrate the predictive potential of the HGSOC-TMI in stratifying patients, even using data collected by using different platforms. Finally, we compared the prognostic performance of the HGSOC-TMI with other two-gene signatures recently identified for ovarian cancer.

## 2. Materials and Methods

### 2.1. Data Retrieval

The list of human matrisome genes [24,25] was retrieved from the website https://web.mit.edu/hyneslab/matrisome/ (accessed on 10 June 2021). The symbols of these 1068 matrisomal genes were inputted into the XENA browser [26] (www.xenabrowser.net, accessed on 10 June 2021) where the study “GDC TCGA Ovarian Cancer” was selected and the normalized RNA-seq data, as well as the associated clinical annotation, were accessed and exported into a spreadsheet for further data curation and analysis. This dataset was used as a “discovery dataset”. Independent HGSOC “validation datasets” (accession numbers: GSE26712 and GSE49997) were accessed by using Phantasus (v.1.11.0, https://artyomovlab.wustl.edu/phantasus/, accessed on 11 June 2021). The “quantile normalization” and “Log_2_” transformations were applied to the data. Moreover, data were collapsed by using “Maximum Median Probe” with “gene symbol” as the collapse field to remove lowly expressed probes and to ensure that only one row per gene was present in the expression matrices.

### 2.2. Construction of the HGSOC-TMI Risk Score

Gene-expression matrix and clinical annotation data for the stage III/IV cohort were imported into RStudio (v1.3.1073, using R, v4.0.2). The Cox proportional hazard model was independently applied to each of the 1068 matrisomal genes, using the “RegParallel” package (v1.8.0). The genes with log-rank *p*-value < 0.01 were used to compute the HGSOC-specific prognostic signature, the HGSOC Tumor Matrisome Index (TMI): HGSOC − TMI = $\sum_{i}Expression(Gene_{i})\cdot Beta_{i}$, where the expression level of each prognostic gene was multiplied by its Cox regression coefficient (Beta). Then a Cox proportional-hazard model was used to estimate the effect of the HGSOC-TMI scores on the patients’ overall survival, using the “coxph” function of the “survival” package (v3.2-11) in R/Bioconductor [27]. The assumption of the Cox proportional hazard was checked by using the scaled Schoenfeld residual test (cox.zph function provided by survival package in R/Bioconductor [27]).

### 2.3. Patient Stratification and Survival Analysis

An optimal cutoff score, computed by using the “maxstat.test” function (“maxstat” package [28]), was used to stratify patients into low- and high-risk groups. Kaplan–Meier (KM) survival curves were generated using the “survival” package to visualize the overall survival (OS) probability associated with each risk group. Log-rank *p*-value ≤ 0.05 was considered statistically significant. The OS time was calculated as the time between the date of surgery and death.

### 2.4. Internal and External Validation of the HGSOC-TMI Risk Score

Internal validation of the HGSOC-TMI was performed by using the penalized Cox model (elastic-net with “Bootstrap validation”) and visualized through the time-dependent AUCs (“hdnom”, v6.0.0 and “rms”, v6.2-0). The external validation was conducted on the HGSOC GEO datasets GSE26712 and GSE49997, where the expression levels of the HGSOC-TMI genes were combined with the betas calculated in the discovery dataset.

### 2.5. Single-Cell Analysis

Data from a single-cell RNA-sequencing analysis [16] of high-resolution dissection of the tumor ecosystem of 15 ovarian tumors were retrieved by using BBrowser (v2.10.13) [29] to identify specific cell types expressing the HGSOC-TMI signature (accession number: EGAS00001004935). Moreover, single-cell gene expression values from a whole-transcriptome analysis [15] (accession number GSE14602) performed across ~11,000 cells from 22 ascites from 11 HGSOC patients were re-analyzed in this study to identify specific cell types expressing the HGSOC-TMI signature. Data were accessed and plots were generated by using BBrowser (v2.10.13) [29] (https://bioturing.com/, accessed on 19 August 2021).

### 2.6. Abundance of Tumor-Infiltrating Immune Cell

The relative estimated abundance of 22 infiltrating immune cell types in each of the HGSOC-TMI risk groups was computed by using the web-based tool CIBERSORTx (https://cibersortx.stanford.edu/, accessed on 23 August 2021). Specifically, the analysis was conducted by selecting the “LM22 signature” as “signature gene file”, with 100 permutations, and without “quantile normalization”. Subsequently, box plots were generated to quantify the differences in infiltrated immune cells between the two risk groups, using “ggplot2” (v3.3.3). Two-sided unpaired two-sample Wilcoxon tests were performed.

### 2.7. Network Analysis

Network analysis of the HGSOC-TMI genes was performed by using NetworkAnalyst (https://www.networkanalyst.ca, accessed on 5 July 2021). Specifically, tissue-specific (ovary) protein–protein interactions (PPIs) analysis was selected.

### 2.8. Machine-Learning Approach for Risk Group Prediction

Using Orange (v3.29.3), the computational classification method recently developed by Belotti et al. [30] and further expanded to test the predictive potential of head and neck carcinoma’s risk score [22], was utilized. The algorithm workflow and the parameters used in this study are shown in the work by Belotti et al. [22]. The classification was also validated by using two independent GEO datasets (GSE26712 and GSE49997).

## 3. Results

### 3.1. Construction and Validation of the HGSOC-TMI

**Figure 1:**
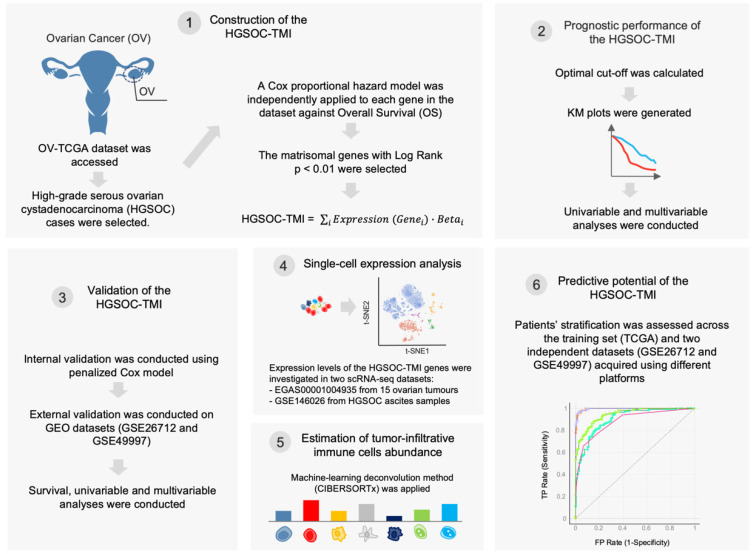
Schematic of the bioinformatics workflow. First, The Cancer Genome Atlas (TCGA) ovarian cancer (OV) dataset was accessed, and the high-grade serous ovarian carcinoma cases were selected. A Cox proportional hazard model was used to identify matrisome-associated prognostic genes. The HGSOC-TMI risk score was computed, and two risk groups were identified by using an optimal cutoff. The overall survival probabilities for each risk group are shown in Kaplan–Meier (KM) plots. The HGSOC-TMI risk score was internally and externally validated by using two independent GEO datasets. A machine-learning-based deconvolution tool (CIBERSORTx) was then used to investigate the abundance of infiltrating immune cells for each risk group. Finally, machine-learning multiclass supervised classification was performed to test the predictive abilities of the HGSOC-TMI across different platforms.

The goal of our study was to identify an ovarian-tissue-specific risk score based on matrisomal components, similar to what has been recently investigated in non-small-cell lung cancer [9] and squamous cell carcinoma of the head and neck [22]. A series of recent web-based tools and open-source software was employed to analyze large datasets from The Cancer Genome Atlas (TCGA) program [31]. Our bioinformatics pipeline is summarized in Figure 1.

**Figure 2:**
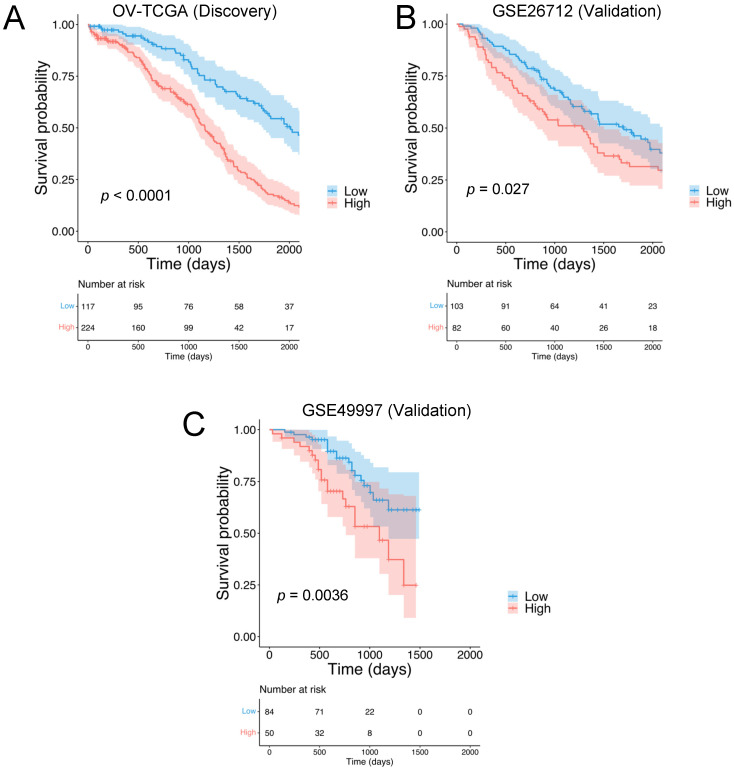
Identification of the HGSOC Tumor Matrisome Index (HGSOC-TMI) and its prognostic ability. Kaplan–Meier plots for the high-grade serous ovarian carcinoma (HGSOC) in the case of (**A**) the discovery set OV-TCGA (N = 341) and the following validation sets: (**B**) GSE26712 (N = 185) and (**C**) GSE49997 (N = 134).

Focusing on RNA sequencing data accessed by using XENA browser, 341 stage III/IV, primary high-grade serous ovarian carcinoma samples were analyzed. A subset of 19 matrisomal genes (*PLXNB2, HMCN2, NDNF, NTN1, TGFBI, CHAD, CLEC5A, PLXNA1, CST9, LOXL4, MMP17, PI3, PRSS1, SERPINA10, TLL1, CBLN2, IL26, NRG4*, and *WNT9A*) with prognostic values were identified by using a Cox proportional hazard model for each matrisomal gene in the dataset against overall survival (OS). The genes with log rank *p* < 0.01 (Supplementary Materials Table S1) were used to compute HGSOC-TMI (Tumor Matrisome Index) risk score. The correlation among the 19 HGSOC-TMI genes was tested, and a low-to-moderate degree of correlation was found, as shown in Supplementary Materials Figure S1. The only high degree of correlation (Pearson’s correlation coefficient of 0.6) was found between the genes *TGFBI* and *CLEC5A*. Nomograms were generated to predict survival probability at 2, 3, and 5 years after surgery (Supplementary Materials Figure S2). The details of these genes are shown in Supplementary Materials Table S2. Using an optimal cutoff, we stratified patients into low- and high-risk groups. Clinical and histopathological information were also downloaded from the XENA browser and added to the gene-expression spreadsheet. The independent effect of each clinical and histopathological factor was assessed by using univariable analysis. Factors that were statistically significant in the univariable analysis underwent multivariable analysis. The results of the univariable and multivariable analyses are shown in Supplementary Materials Figure S3. The risk group was an independent prognostic factor, even after the adjustment (HR = 2.8, 95% CI = 2.1–3.8, *p* < 0.001). Kaplan–Meier (KM) curves were generated to test the prognostic values of the HGSOC-TMI. The low-risk group showed a longer survival probability, as shown in Figure 2A.

The prognostic value of the HGSOC-TMI was internally validated by using machine-learning-based algorithms. Specifically, the internal validation was performed by using the penalized Cox model. In Supplementary Materials Figure S3C the time-dependent AUC (area under the receiver operating characteristic (ROC) curve) is shown. External validation was performed on two independent Gene Expression Omnibus (GEO) datasets: GSE26712 and GSE49997 (Figure 2B,C).

### 3.2. Single-Cell Analyses

**Figure 3:**
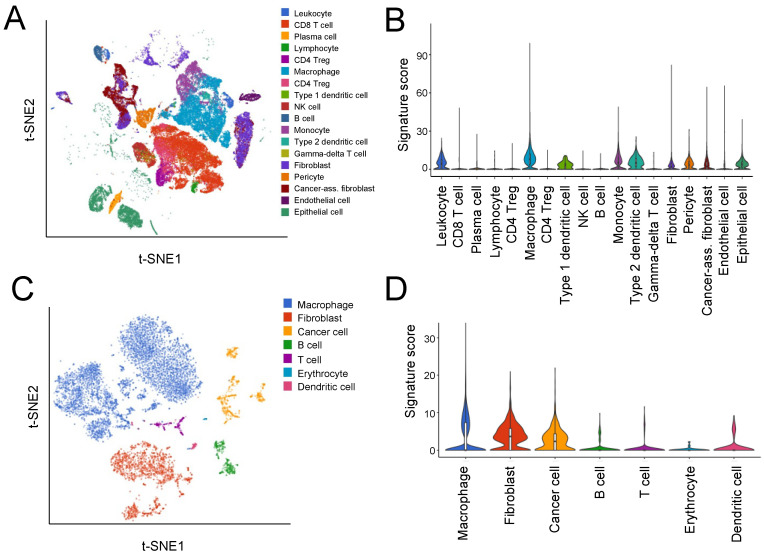
Expression of the HGSOC-TMI signature at the single-cell level. (**A**) A t-SNE plot from the scRNA-seq study by Hornburg et al. [16] (EGAS00001004935 dataset) on 15 ovarian cancer patients colored by cell type. (**B**) Relative expression of the HGSOC-TMI genes across different cell types. On the *y*-axis, the average expression of the HGSOC-TMI genes. (**C**) A t-SNE plot of 9609 droplet-based scRNA-seq profiles from eight samples of ovarian cancer ascites from Izar et al. [15], colored by cell type (GSE14602 dataset). (**D**) Relative expression of the HGSOC-TMI genes across different cell types.

In order to identify the specific cell types expressing the HGSOC-TMI signature, we accessed a single-cell RNA-seq dataset generated in the recent study by a Hornburg et al. [16] where the authors conducted a high-resolution dissection of the tumor ecosystem of 15 ovarian tumors (accession number: EGAS00001004935). Figure 3A shows the t-SNE plot of 40,539 tumor cells, 35,296 stromal cells, 15,049 immune cells, and 2334 stromal/immune cells analyzed in the study by Hornburg et al. [16]. The mean expression of the HGSOC-TMI genes across different cell types is shown in Figure 3B. A higher expression of these genes was found in macrophages, type 1 dendritic cells, monocytes, type 2 dendritic cells, leukocytes, pericytes, epithelial cells, fibroblasts, and cancer-associated fibroblasts, as compared with other immune cells investigated in this study. Specifically, the main contribution in terms of mean expression level and proportion of cells expressing the specific gene was found to be associated with *TGFBI, PLXNB2,* and *CLEC5A,* as shown in Supplementary Materials Figure S5A. Furthermore, a small portion of macrophages was found to have high expression of *CBLN2* and *PRSS1*. Moreover, we investigated the expression of the HGSOC-TMI genes in the scRNA-seq dataset from a recent paper by Izar et al. [15] where the authors analyzed ascites samples from HGSOC patients (accession number: GSE146026). Specifically, 35,957 high-quality cell profiles from a set of 22 ascites samples were investigated (Figure 3C). The mean expression of the HGSOC-TMI genes across different cell types found in the ascites samples is shown in Figure 3D. Specifically, a high proportion of fibroblasts, macrophages, and cancer cells were found to exhibit high expression of *TGFBI,* whereas a high proportion of cancer cells and fibroblasts were found to have high expression of *PLXNB2,* as shown in Supplementary Materials Figure S5B. High expressions of these two genes were also found in smaller proportions of dendritic cells and T cells.

### 3.3. Association between Risk Groups and Tumor-Infiltrating Immune Cells

**Figure 4:**
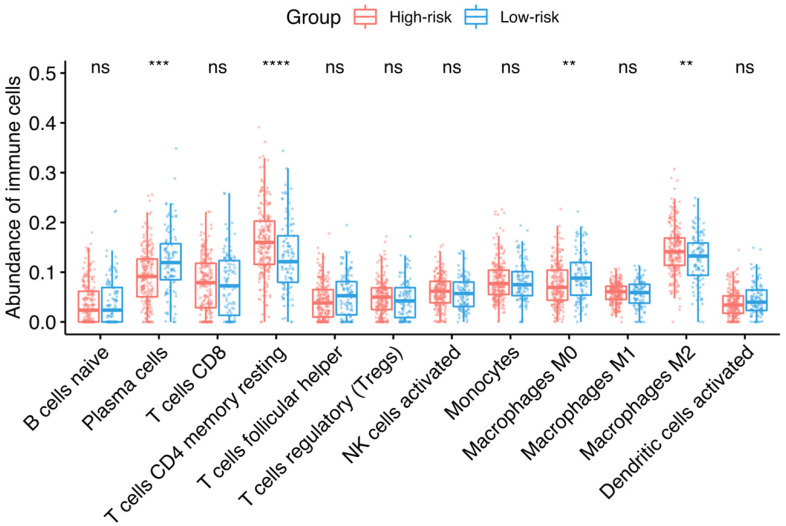
Infiltrating immune cells and HGSOC-TMI risk group. Median percentages of infiltrating immune cells were estimated between each risk group in the OV-TCGA dataset, using CIBERSORTx. The size of the boxes corresponds to the interquartile range (IQR = Q3 − Q1). The whiskers indicate the range from Q1 + 1.5 × IQR to Q3 − 1.5 × IQR. A two-sided unpaired two-sample Wilcoxon test was performed between the two groups: **** *p* ≤ 0.0001; *** *p* ≤ 0.001; ** *p* ≤ 0.01; ns, not significant.

The role of tumor-infiltrating immune cells was investigated in the discovery dataset across the identified risk groups. The machine-learning tool CIBERSORTx was used to deconvolve the relative abundance of 22 types of immune cells from TCGA data. Figure 4 shows that statistically significant lower abundances of plasma cells and M0 macrophages were found in the high HGSOC-TMI group, while a lower abundance of CD4+ memory-resting T cells and M2 macrophages was found in the low HGSOC-TMI group.

### 3.4. Machine-Learning Approach for Risk Group Prediction

**Figure 5:**
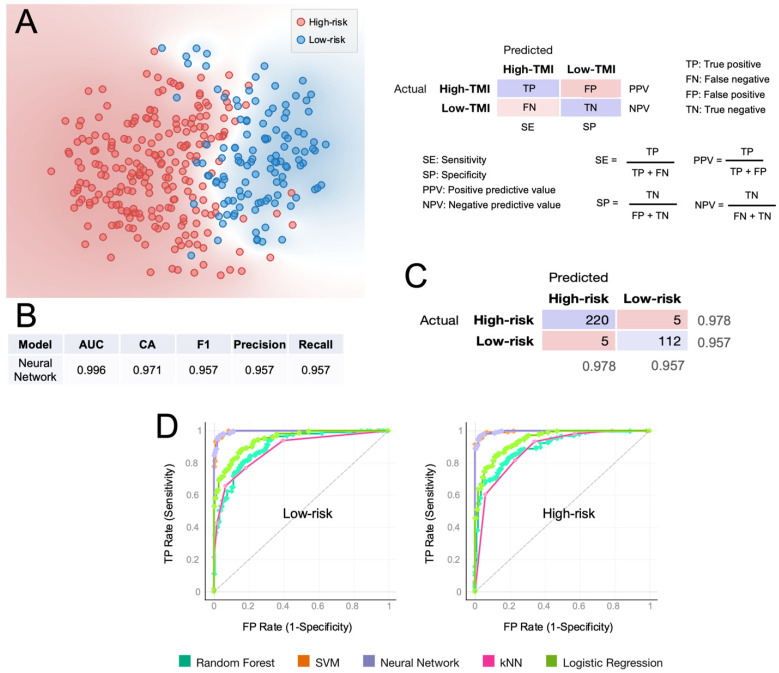
Computational classification of the HGSOC-TMI risk group at the patient level. (**A**) A t-SNE plot of two risk groups for the training set (TCGA). (**B**) Results of the classification model evaluation using 10-fold cross-validation. (**C**) Confusion matrix of the neural-network model, which had the best scored in the classification. (**D**) Receiver operating characteristic (ROC) curves for two HGSOC-TMI risk groups. AUC = area under the curve. CA = classification accuracy.

We sought to assess the predictive value of the HGSOC-TMI in discriminating between the risk groups, using a supervised machine-learning approach [30]. The t-distributed Stochastic Neighbor Embedding (t-SNE) plot in Figure 5A shows a clear separation between the two risk groups. Multiple computational predictive models were then trained on the OV-TCGA dataset (Figure 5). The predictive performance of each model was tested through 10-fold cross-validation. The best predictive model was the neural network, which achieved a classification accuracy of 97.1% and an AUC of 0.996, which is the area under the receiver operating characteristic (ROC) curve. The confusion matrix in Figure 5C shows the number of patients that are correctly predicted for each risk group. In Figure 5D, the ROC curves for each predictive model are shown.

**Figure 6:**
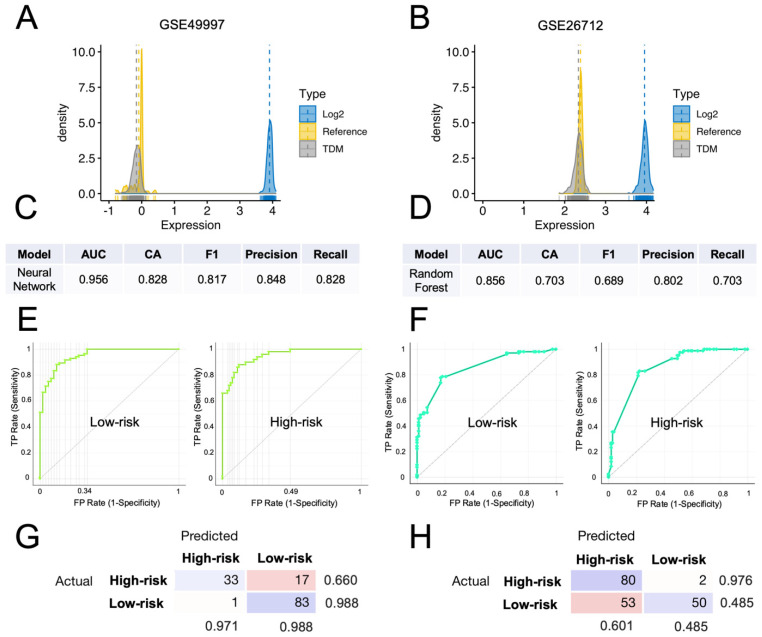
External validation of the computational classification of the HGSOC-TMI risk group. (**A**,**B**) Density plots of the TDM and log_2_-transformed training data (TCGA) against the validation datasets (GSE49997 and GSE26712). (**C**) Results of the neural-network model, which had the best score in the classification of the risk groups in the GSE49997 dataset. (**D**) Results of the random-forest model, that had the best score in the classification of the risk groups in the GSE26712 dataset. (**E**) Receiver operating characteristic (ROC) curves for two HGSOC-TMI risk groups for the GSE49997 and (**F**) GSE26712 datasets. (**G**,**H**) Confusion matrixes showing the number of patients correctly classified by using the best predictive model for each dataset.

To demonstrate the predictive potential of the HGSOC-TMI in stratifying patients in independent datasets across different platforms, we transformed the TCGA data to make them comparable with the microarray ones. Specifically, we applied the cross-platform TDM transformation [32] to the RNA-seq data, as recently shown [12,22]. In Figure 6A,B, the comparison between TDM and logarithmic transformation is shown for both validation datasets. The TDM transformation best fitted the distribution of the reference microarray data (GSE49997 and GSE26712). Multiple computational predictive models were then trained on the TDM-transformed OV-TCGA dataset and tested on both GEO datasets. The best predictive model on the GSE49997 dataset was the neural-network model, which achieved a classification accuracy of 82.8.1% and an AUC of 0.956 (Figure 6E), whereas the random-forest model achieved an accuracy of 70.3% and AUC of 0.856 (Figure 6F) on the GSE26712 dataset. The confusion matrixes in Figure 6G,H show the number of patients that are correctly predicted for each risk group, in each dataset.

### 3.5. Comparison with Other Prognostic Gene Signatures

We sought to compare the prognostic performance of the HGSOC-TMI with other two-gene signatures previously identified for ovarian cancer. The first included the Galectin 1, 3, and 7 genes (*LGALS1, LGALS3*, and *LGALS7*) that were previously investigated by Schulz et al. [33], whereas the second signature included five immune microenvironment genes (*ETV7*, *GBP4*, *CXCL9*, *CD3E*, and *TAP1*) that were recently found by Huo et al. [34] to significantly correlate with prognosis. Using the OV-TCGA dataset, we applied the Cox proportional hazard model to these two-gene panels and found that none of the genes had a statistically significant log-rank *p*-value in HGSOC, as shown in Supplementary Materials Table S3.

## 4. Discussion

The development of novel methods to identify patients in need of further adjuvant treatment after surgical resection is an important goal in cancer-treatment management. In the pursuit of such a goal, we analyzed RNA sequencing data from a large public dataset of high-grade serous ovarian carcinoma and successfully identified a set of matrisomal genes with prognostic value. Specifically, by computing the HGSOC-TMI risk score, we managed to stratify patients into risk groups. Furthermore, using supervised machine-learning-based methods, we managed to demonstrate the high accuracy and robustness of the HGSOC-TMI in risk stratification, even across different platforms (microarrays and RNA-seq), with important clinical implications. Having identified the role of specific ECM components in HGSOC progression, we believe that future investigations of the HGSOC-TMI in both early stage and metastatic ovarian cancers could provide potential insights into novel druggable molecular targets. Prospective randomized interventional studies will better elucidate the clinical value of our findings in HGSOC progression and metastasis. The KEGG signaling network analysis revealed that the HGSOC-TMI genes are mostly associated with “axon guidance” and “proteoglycans in cancer” pathways (Supplementary Materials Figure S4), a finding that is in agreement with previous studies that found evidence for the involvement of both signaling pathways in the progression of ovarian cancer [35,36,37]. Most of the 19 HGSOC-TMI genes were previously found to have a role in OC. Specifically, *TGFBI,* which encodes an RGD-containing protein that binds to type I, II, and IV collagens that are involved in modulating cell adhesion [38], was previously shown to be frequently methylated in ovarian cancer, and this could be leveraged as an epigenetic biomarker for discriminating ovarian cancer from non-cancer or borderline tumors [39]. This gene was found to act both as a tumor promoter or suppressor depending on the tumor microenvironment [40]. Moreover, it was recently shown to elicit an immunosuppressive microenvironment in advanced HGSOC and mediate tumor-promoting actions of TGFβ and, hence, represents a potential drug target [41]. In another recent work, the authors demonstrated that *TGFBI* secreted by tumor-associated macrophages (TAM) promotes HGSOC‘s progression [42]. *PLXNB2* was previously associated with cell proliferation, invasion, and decreased phosphorylation of AKT and ERK1/2 in ovarian cancer cells [43]. Moreover, the authors demonstrated the existence of an association between miR-126-3p overexpression and *PLXNB2* downregulation on the cell-growth viability, cell colony, and cell invasion, suggesting that miR-126-3p affects ovarian cancer’s progression by direct regulation of *PLXNB2*. Dysregulation of *PLXNs* was associated with the existence of immune subtypes in various cancer types’ characterized by an anomalous level of immune and stromal-cell infiltrates in the TME, which is frequently associated with patient survival and drug responses [44]. *PLXNB2* encodes a cell surface receptor that plays an important role in controlling changes in the actin cytoskeleton, axon guidance, invasive growth, and cell migration [45,46]. Moreover, this gene was shown to negatively regulate macrophage motility, as well as Rac and Cdc42 activation [47]. The peptidase inhibitor 3 (*PI3*) is a serine protease inhibitor with known anti-inflammatory, antimicrobial, and immune-modulatory properties [48] and was shown to be downregulated during ovarian tumorigenesis, while its residual expression is predictive of recurrence [49]. Two recent studies independently identified *PI3* as a potential prognostic biomarker for OC [50,51]. *PLXNA1* encodes a co-receptor for semaphorins and plays a role in axon guidance, invasive growth, and cell migration [52]. A recent study found that *PLXNA1* was upregulated in many cancer types, and its increased expression was associated with poor prognostics and correlated with more aggressive subtypes of immune infiltrates [44]. *CLEC5A* is closely related to the abundance of infiltrated immune cells and the expression of immune checkpoints in the OC microenvironment [53]. Specifically, high expression of *CLEC5A* in OC cells was linked with increased polarization of M2 macrophages. This gene encodes a member of the C-type lectin/C-type lectin-like domain (CTL/CTLD) superfamily that is involved in cell adhesion, glycoprotein turnover, cell–cell signaling, inflammation, and immune response [54]. In recent work, using immunostaining and fluorescent imaging, the authors revealed that the gene *PRSS1* was overexpressed in the endoplasmic reticulum of a high-grade serous ovarian cancer cell line [55]. Moreover, overexpression of this gene predicts platinum resistance in OC patients [56]. Downregulation of *HMCN2* was recently associated with the inhibition of cell invasion [57]. This gene encodes a protein that belongs to the fibulin, which regulates tissue adhesion and cell migration [58]. Downregulation of *NDNF* was associated with longer survival [59]. This protein is involved in cell adhesion, the extracellular matrix in assembly, and the regulation of growth factors’ activity [60]. The expression of *NTN1* was strongly upregulated in malignant ovarian tumors when compared with benign tumors [61]. This gene is thought to be involved in axon guidance and cell migration during development and tumorigenesis by regulating apoptosis [62,63]. The role of *NTN1* in cancer and immunomodulation has been reviewed by Bruikman et al. [64]. Overexpression of *LOXL4* was associated with worse progression-free survival (PFS) in OC patients [65]. This gene plays a role in tumor suppression, cell-growth control, and chemotaxis [66]. *SERPINA10,* which encodes a protein that belongs to the serpin family, was recently found to be positively correlated with overall survival OS and PFS in HGSOC [67]. Moreover, using immunohistochemistry (IHC), the authors demonstrated that HGSOC patients with high *SERPINA10* expression had longer platinum-free interval (PFI) than the patients with low expression. *TLL1,* which encodes an astacin-like, zinc-dependent metalloprotease that belongs to the peptidase M12A family [68], was recently identified as a risk factor in OC [69]. The prognostic role of *IL26* was recently demonstrated in OC [70]. This gene encodes a protein with proinflammatory functions involved in mucosal immunity; activates MAPK1/3, JUN, STAT1 and STAT3, and AKT; and induces the expression of ICAM1, IL-8, TNF-alpha, SOCS3 and the secretion of IL-8 and IL-10 [71]. *NRG4*, which is involved in the activation of type-1 growth factor receptors [72], was found to be significantly associated with OS in OC patients [73]. A transcriptome analysis of ascites-derived ovarian cancer cells and TAMs revealed that *WNT9A* is differentially expressed in tumor cells and TAMs [36], and its expression levels are correlated with patient survival [74].

Upon stratification, differential expression between the two risk groups was found for most of the HGSOC-TMI genes, as shown in Supplementary Materials Figure S6. Moreover, each risk group was found to be characterized by different patterns of tumor-infiltrating immune cells by using machine-learning-based deconvolution methods. Specifically, a higher abundance of M2-like macrophages was found in the high-risk group. This result is in accordance with previous works in the literature that demonstrated the pro-tumoral role of the M2 phenotype [75,76,77]. To date, however, no reports have elucidated the role of resting macrophages (M0) in HGSOC. These cells, also known as “uncommitted” macrophages, become polarized as a consequence of cytokine stimuli [78,79]. Their role appears to be tissue-dependent; for instance, Tekin et al. [80] linked them with anti-tumorigenic activities mediated by TNF-α in pancreatic cancer, whereas the opposite was found in breast cancer by Ali et al. [81]. Our analysis showed a higher abundance of M0-like macrophages in the HGSOC-TMI high-risk group. On the other hand, in a recent meta-analysis conducted on 10 independent studies with 1815 patients, Liu et al. [77] found a positive association between M0 macrophages infiltration and positive prognosis after platinum-based chemotherapy. Our findings are in agreement with the previous literature, where TAMs were shown to play a key role in the microenvironment of ovarian tumors, as they are among the main regulators of the interactions between the immune response and cancer cells [82,83,84,85]. Furthermore, we found that tumors in the HGSOC-TMI high-risk group were associated with increased CD4+ resting-memory T-cells’ infiltration, which is consistent with a prior work by Bi et al. [86] that found these cells to exhibit a higher infiltration in low-tumor mutation burden (TMB) group, typically associated with lower OS in OC patients. A higher abundance of plasma cells was found in the low-risk group, and this finding is in agreement with a systematic review by Wouters and Nelson [87] in which the authors analyzed 21 studies and found that the majority of them reported plasma cells to be associated with a positive prognosis.

The single-cell analysis demonstrated the expression of the HGSOC-TMI genes in cancer cells, as well as in immune cells in HGSOC cancer tissue and ascites samples.

Moreover, our results showed a superior prognostic value of the HGSOC-TMI risk score compared with the two gene signatures previously reported by Schulz et al. [33] and Huo et al. [34].

One of the limitations of this study is that publicly available algorithms could be modified over time or no longer maintained. This might affect the reproducibility of this study. However, on the other hand, novel efficient algorithms and web-based interfaces are constantly developed; therefore, the analysis of sequencing data will likely be further simplified in the future. Moreover, further validation of the HGSOC-TMI signature by using data from large cohorts is required in the future. This will allow for the identification of a universal cutoff value (as shown in non-small-cell lung cancer [9]) for patient stratification and will further improve the clinical utility of the HGSOC-TMI. Given the small number of genes in the HGSOC-TMI, quantification of the expression levels could be conducted through RT-PCR directly on postoperative biopsy specimens.

Molecular diagnostics, especially transcriptomics, is becoming one of the key drivers of personalized oncology, as it enables tumor phenotyping with unprecedented precision, with important clinical results (as recently reviewed by Buzdin et al. [88]). Direct high-throughput measurements of the expression levels of drug targets and specific molecular pathways enable the monitoring of the efficacy of personalized molecular therapies. In the future, single-cell studies focusing on transcriptomic changes at different tumor stages and at multiple time points during cancer progression or drug treatment will further elucidate the role of the HGSOC-TMI genes, as well as their interactions with infiltrating immune cells, and provide novel mechanistic insights into this disease and the efficacy of the treatments.

## 5. Conclusions

In conclusion, using bioinformatics approaches, a 19-gene TME-related HGSOC prognostic genetic panel was identified and further validated in two independent RNA-seq datasets. Moreover, using machine-learning-based methods, specific patterns of infiltrating immune cells were found to characterize different risk groups. The predictive potential of the HGSOC-TMI risk score was demonstrated across different platforms. We confirmed the expression of the HGSOC-TMI genes in both cancer and immune cells in scRNA-seq data from HGSOC tissue, as well as ascites, samples. Finally, improved prognostic performance of the HGSOC-TMI was found compared with two previously identified ovarian cancer gene panels. This study might have important implications in the prognosis and personalized adjuvant treatments of HGSOC.

## Data Availability

The data analyzed in this study were obtained from TCGA, using the XENA browser, accessing the “GDC TCGA Ovarian Cancer” study. The validation dataset was obtained from GEO (Gene Expression Omnibus), under the accession codes GSE26712 and GSE49997, accessed by using Phantasus. The scRNA-seq dataset analyzed in this study is available from GEO (Gene Expression Omnibus), under the accession code GSE14602; and BBrowser, under the accession code EGAS00001004935.

## Supplementary Materials

The following are available online at https://www.mdpi.com/article/10.3390/cancers14020404/s1. Figure S1: Correlation matrix of the 19 HGSOC-TMI genes. Figure S2: Nomogram to predict survival probability at 2, 3, and 5 years after surgery for stage III/IV high-grade serous ovarian carcinoma (HGSOC) patients based on the expression levels of the HGSOC-TMI genes derived from the TCGA dataset. Figure S3: Univariable analysis, multivariable analysis, and internal validation of the Cox model. Figure S4: Ovary-specific protein–protein interactions (PPIs) network analysis via NetworkAnalyst and data from the DifferentialNet database. Figure S5: Expression of the HGSOC-TMI genes at the single-cell level. Figure S6: Expression levels for each HGSOC-TMI gene as a function of the risk group. Table S1: Results of the gene-selection step. Table S2: Details of the 19 genes composing the HGSOC-TMI. Table S3: Calculation of the hazard ratio (HR) for two previously published prognostic gene panels.
