# Supplementary material for: The Role of the Extracellular Matrix and Tumor-Infiltrating Immune Cells in the Prognostication of High-Grade Serous Ovarian Cancer
Source: Cancers (Basel). 2022 Jan 14;14(2):404. doi: 10.3390/cancers14020404 (PMC8773831; doi:10.3390/cancers14020404)
Supplement: Supplementary file 1 [file cancers-14-00404-s001.zip › cancers-1511750-supplementary.pdf]

Supplementary materials

The Role of the Extracellular Matrix and Tumor-Infiltrating Immune Cells in the Prognostication of High-Grade Serous Ovarian Cancer

Table S1. Results of the gene selection step. Cox proportional hazard model was applied to the 1068 matrisomal genes. The list of 19 genes was selected based if Log Rank $p < 0.01$.

Gene	Beta	Std Error	Z	P	LRT	Wald	Log-Rank	HR	HR_{lower}	HR_{upper}
HMCN2	0.14519241	0.03403185	4.26636831	1.99E-05	3.47E-05	1.99E-05	1.93E-05	1.15626203	1.08165385	1.23601639
PI3	0.10484167	0.02888382	3.62977158	0.00028367	0.00027718	0.00028367	0.00028389	1.11053476	1.04941243	1.17521712
SERPINA10	-0.0589842	0.01753317	-3.364149	0.0007678	0.00076552	0.0007678	0.00070218	0.94272167	0.91087594	0.97568078
CBLN2	-0.0540807	0.01716264	-3.1510712	0.00162673	0.00193525	0.00162673	0.00155656	0.94735564	0.91601835	0.97976498
NTN1	0.1579572	0.05095089	3.10018514	0.001934	0.00179032	0.001934	0.00195219	1.17111607	1.05981598	1.29410472
MMP17	0.13420497	0.04397625	3.05176024	0.00227504	0.00251033	0.00227504	0.00237567	1.1436272	1.0491844	1.24657131
CST9	-0.0359263	0.01236334	-2.905874	0.00366229	0.00419052	0.00366229	0.00350169	0.96471138	0.94161573	0.98837351
PLXNA1	0.26492022	0.09136019	2.8997336	0.0037348	0.00347047	0.0037348	0.00372179	1.30332699	1.08965108	1.55890383
IL26	-0.0436794	0.01515176	-2.8827939	0.00394165	0.00330757	0.00394165	0.00370678	0.9572608	0.92925107	0.9861148
PRSS1	-0.0936313	0.03257514	-2.8743188	0.004049	0.00400906	0.004049	0.00420561	0.91061841	0.85429609	0.97065397
NRG4	-0.1205386	0.04222589	-2.8546124	0.00430894	0.00452003	0.00430894	0.00427089	0.88644291	0.81603357	0.96292733
TGFBI	0.15783573	0.05603467	2.81675121	0.00485121	0.00454059	0.00485121	0.00482519	1.17097383	1.04918093	1.3069049
CHAD	0.13661566	0.04868438	2.80615003	0.00501373	0.00424249	0.00501373	0.00517656	1.14638746	1.04205637	1.26116423
WNT9A	0.16577986	0.05988967	2.7680877	0.00563863	0.00501445	0.00563863	0.00559548	1.18031324	1.04958859	1.32731946
TLL1	0.09991732	0.03639466	2.74538406	0.00604401	0.00565613	0.00604401	0.00611775	1.10507954	1.02899757	1.18678686
NDNF	0.08379905	0.03132933	2.67477952	0.00747784	0.00501505	0.00747784	0.00810833	1.08741035	1.02264733	1.15627473
CLEC5A	0.12685922	0.04746962	2.67242953	0.00753042	0.00657389	0.00753042	0.00748545	1.13525719	1.03439888	1.2459496
PLXNB2	0.26206283	0.10019138	2.6156225	0.0089065	0.00861612	0.0089065	0.00896671	1.2996082	1.06789704	1.5815958
LOXL4	0.11645301	0.04527105	2.57235058	0.01010105	0.00968031	0.01010105	0.00998872	1.12350472	1.02811125	1.22774928

Table S2. Details of the 19 genes composing the HGSOC-TMI.

Gene Symbol	Division	Category	Gene Name	Synonyms	HGNC_IDs
HMCN2	Core matrisome	ECM Glycoproteins	hemicentin 2	DKFZp434P0216|FLJ23816|FLJ46139|FLJ46844	21293
NDNF	Core matrisome	ECM Glycoproteins	chromosome 4 open reading frame 31	FLJ23191|C4orf31	26256
NTN1	Core matrisome	ECM Glycoproteins	netrin 1	NTN1L	8029
TGFBI	Core matrisome	ECM Glycoproteins	transforming growth factor, beta-induced, 68kDa	BIGH3|CDB1|CDG2|CDGG1|CSD|CSD1|CSD2|CSD3|EBMD|LCD1	11771
CHAD	Core matrisome	Proteoglycans	chondroadherin	SLRR4A	1909
CLEC5A	Matrisome-associated	ECM-affiliated Proteins	C-type lectin domain family 5, member A	CLECSF5|MDL-1|MDL1|MGC138304	2054
PLXNA1	Matrisome-associated	ECM-affiliated Proteins	plexin A1	DKFZp761P19121|NOV|NOVP|PLEXIN-A1|PLXN1	9099
PLXNB2	Matrisome-associated	ECM-affiliated Proteins	plexin B2	KIAA0315|MM1|Nbla00445|PLEXB2|dJ402G11.3	9104
CST9	Matrisome-associated	ECM Regulators	cystatin 9 (testatin)	CLM	13261
LOXL4	Matrisome-associated	ECM Regulators	lysyl oxidase-like 4	FLJ21889|LOXC	17171
MMP17	Matrisome-associated	ECM Regulators	matrix metallopeptidase 17 (membrane-inserted)	MT4-MMP	7163
PI3	Matrisome-associated	ECM Regulators	peptidase inhibitor 3, skin-derived	ESI|MGC13613|SKALP|WAP3|WFDC14|cementoin	8947
PRSS1	Matrisome-associated	ECM Regulators	protease, serine, 1 (trypsin 1)	TRP1|TRY1|TRY4|TRYP1	9475
SERPINA10	Matrisome-associated	ECM Regulators	serpin peptidase inhibitor, clade A (alpha-1 antiproteinase, antitrypsin), member 10	PZI|ZPI	15996
TLL1	Matrisome-associated	ECM Regulators	tolloid-like 1	ASD6|TLL	11843
CBLN2	Matrisome-associated	Secreted Factors	cerebellin 2 precursor	-	1544
IL26	Matrisome-associated	Secreted Factors	interleukin 26	AK155|IL-26	17119
NRG4	Matrisome-associated	Secreted Factors	neuregulin 4	DKFZp779N0541|DKFZp779N1944|HRG4	29862
WNT9A	Matrisome-associated	Secreted Factors	wingless-type MMTV integration site family, member 9A	MGC138165|MGC141991|WNT14	12778

Table S3. Calculation of the Hazard Ratio (HR) for two previously published prognostic gene panels. The Cox proportional hazard model was applied to the two gene panels proposed by (Schulz *et al*, 2017) and (Huo *et al*, 2021).

Gene	Beta	Std Error	Z	P	LRT	Wald	LogRank	HR	HRlower	HRupper
CXCL9	-0.0466306	0.02491284	-1.8717505	0.06124114	0.06403345	0.06124114	0.06114808	0.95443989	0.90895574	1.00220007
TAP1	-0.0865703	0.05105677	-1.6955703	0.08996729	0.09281314	0.08996729	0.08991567	0.91707104	0.82974258	1.01359061
GBP4	-0.0580692	0.04001591	-1.4511518	0.1467376	0.14961896	0.1467376	0.14649667	0.94358469	0.87240728	1.02056927
LGALS7	-0.0136028	0.01241159	-1.0959796	0.27308769	0.27541269	0.27308769	0.27272105	0.98648926	0.96278119	1.01078112
ETV7	-0.0419544	0.04145967	-1.0119318	0.31157068	0.31452243	0.31157068	0.31142873	0.95891355	0.88407461	1.04008777
CD3E	-0.0323792	0.03404878	-0.9509663	0.34162149	0.34532001	0.34162149	0.34156591	0.96813935	0.90563979	1.0349521
LGALS1	-0.0114334	0.06072881	-0.1882706	0.85066453	0.85070583	0.85066453	0.85066402	0.98863166	0.87769184	1.11359423
LGALS3	0.00671813	0.06583066	0.10205166	0.91871567	0.91871734	0.91871567	0.91871562	1.00674075	0.88487614	1.14538847

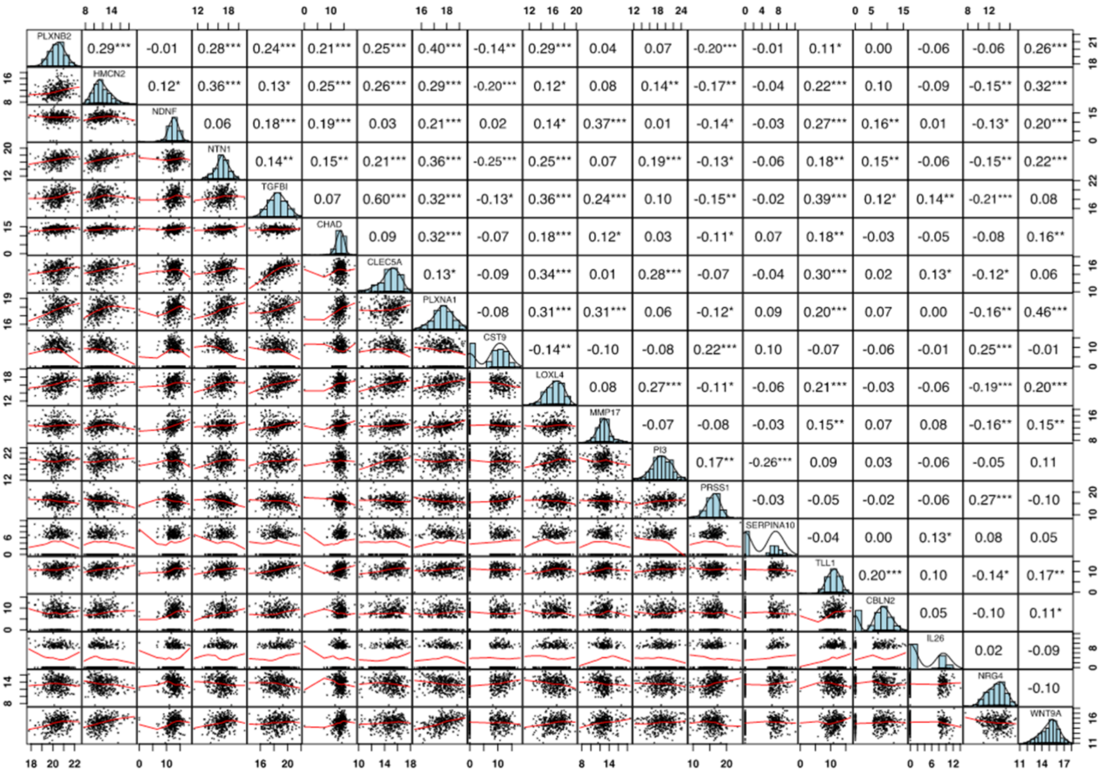

Figure S1. Correlation matrix of the 19 HGSOC-TMI genes. The distribution of each gene's expression level is shown on the diagonal. On the bottom of the diagonal, the bivariate scatter plots with a fitted line are displayed. On the top of the diagonal, the value of the Pearson correlations plus the significance levels are shown. *** $p \leq 0.001$; ** $p \leq 0.01$; * $p \leq 0.05$.

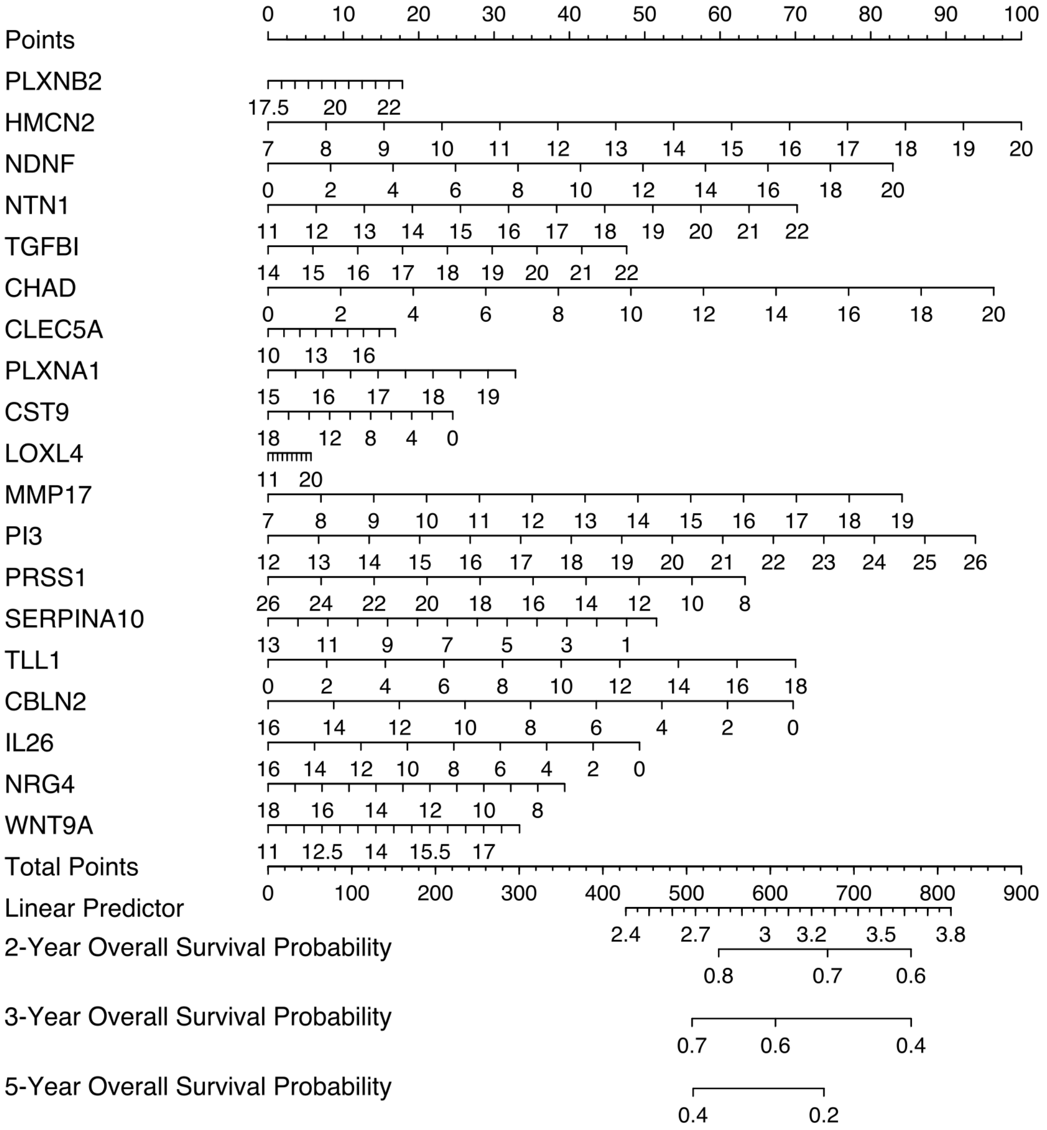

Figure S2. Nomogram to predict survival probability at 2, 3, and 5-year after surgery for stage III-IV, high-grade serous ovarian carcinoma (HGSOC) patients based on the expression levels of the HGSOC-TMI genes derived from the TCGA dataset.

A

Covariates	beta	HR (95% CI)	Wald test	*p* value
Risk group	1	2.8 (2-3.8)	42	9.70E-11
Age	0.021	1 (1-1)	11	0.0011
Lymphatic invasion	0.34	1.4 (0.82-2.4)	1.5	0.22
Venous invasion	-0.16	0.85 (0.44-1.6)	0.24	0.62
Grade	0.0047	1 (0.67-1.5)	0	0.98

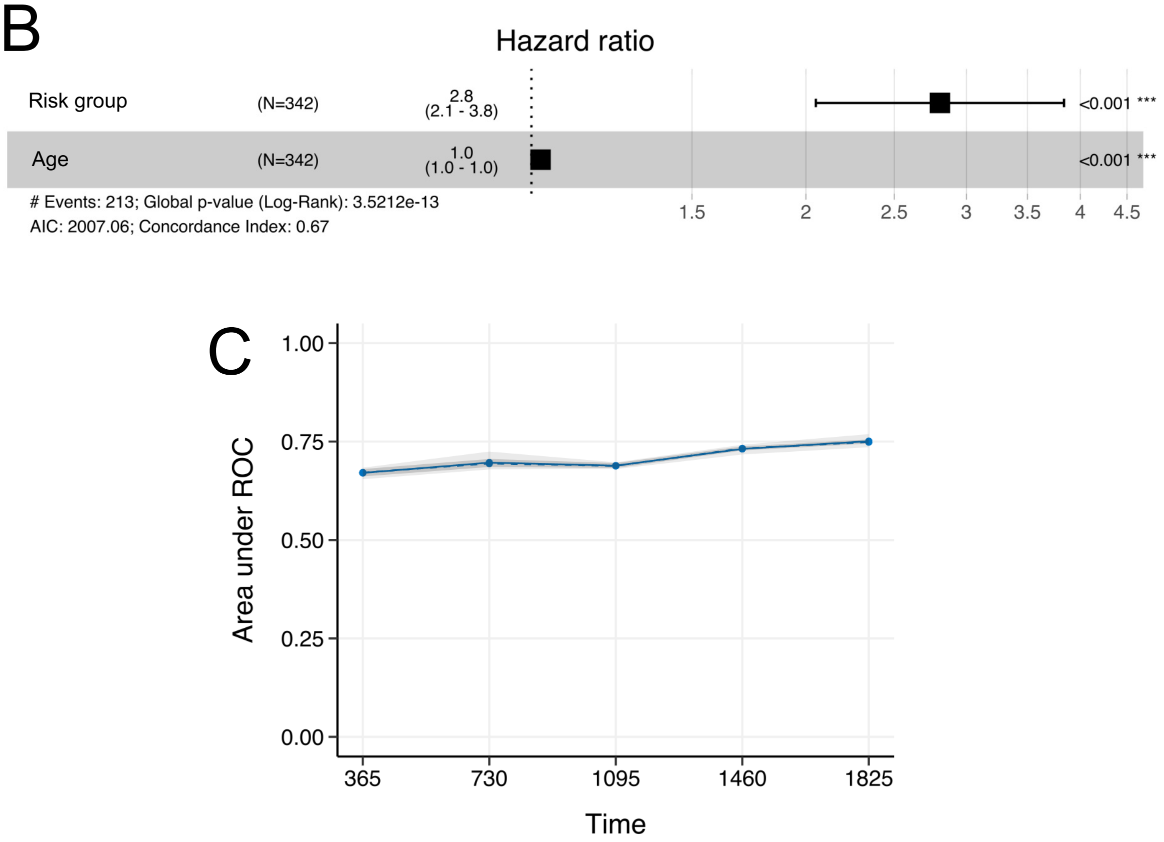

Figure S3. Univariable, multivariable analysis, and internal validation of the Cox model. (**A**) Univariable analysis: the prognostic factor "risk group" was compared with other clinical covariates using the Cox proportional hazard model. (**B**) The forest plot depicts the result of the multivariable analysis of the covariates that were statistically significant at the univariable analysis. (**C**) The prognostic value of the HGSOC-TMI was internally validated using the penalized Cox model (elastic-net with "Bootstrap validation" penalty trade-off parameter α = 0.05, regularization parameter λ = 1.763886) and visualized using the time-dependent AUC.

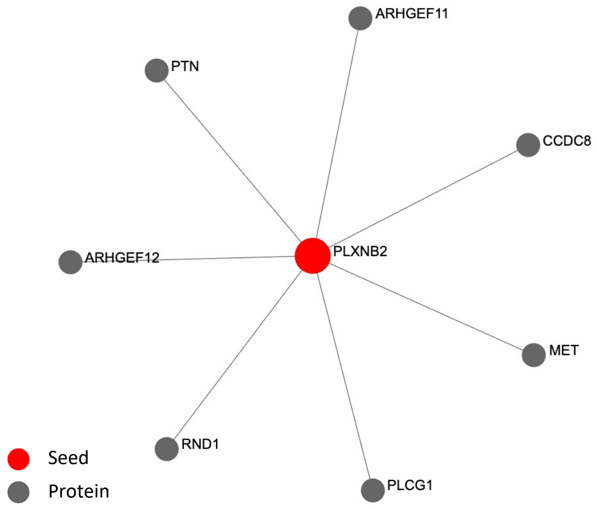

Pathway	Total	Expected	Hits	P.Value	FDR
Axon guidance	181	0.14	5	3.91E-08	1.24E-05
Pathways in cancer	530	0.411	4	0.000292	0.0346
Proteoglycans in cancer	201	0.156	3	0.000326	0.0346
Epithelial cell signaling in Helicobacter pylori infection	68	0.0527	2	0.00112	0.0887
EGFR tyrosine kinase inhibitor resistance	79	0.0613	2	0.0015	0.0956
Vascular smooth muscle contraction	132	0.102	2	0.00414	0.22
Rap1 signaling pathway	206	0.16	2	0.00986	0.448
Ras signaling pathway	232	0.18	2	0.0124	0.493
MicroRNAs in cancer	299	0.232	2	0.0201	0.712
Malaria	49	0.038	1	0.0374	0.778

Figure S4. Ovary-specific protein-protein interactions (PPI) network analysis via NetworkAnalyst and data from the DifferentialNet database. The network has 8 nodes, 7 edges, and 1 seed. On the right, the top 10 scores of the KEGG (Kyoto Encyclopedia of Genes and Genomes) enrichment analysis are shown.

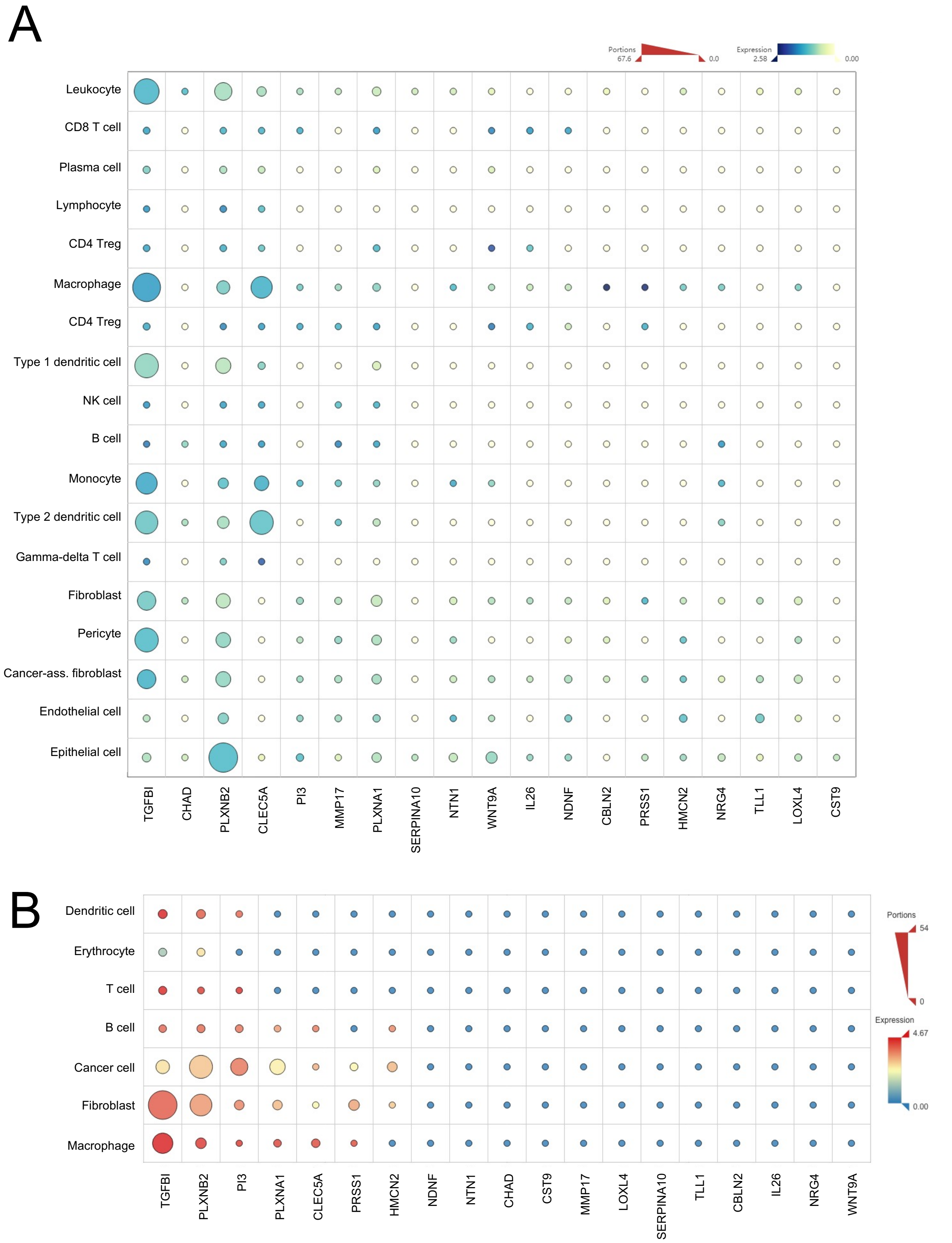

Figure S5. Expression of the HGSOC-TMI genes at the single-cell level. (**A**) Heatmap of the expression level for each of the genes in the HGSOC-TMI signature across different cell types in the scRNA-seq study by Hornburg et al. [1] (EGAS00001004935 dataset). (**B**) Heatmap of the expression level for each of the genes in the HGSOC-TMI signature across different cell types in the scRNA-seq study from Izar et al. [2] (GSE14602 dataset). In both dot plots, the dot size represents the proportion of cells within the respective cell type expressing the specific gene and the color represents the average gene expression level within each cell type.

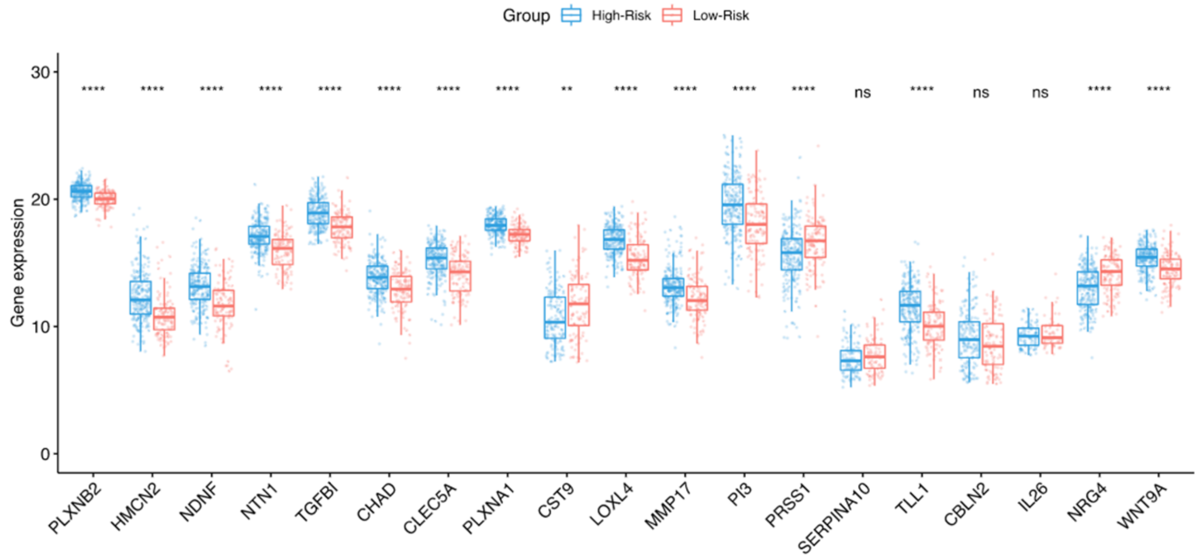

Figure S6. Expression levels for each HGSOC-TMI gene as a function of the risk group. Specifically, 117 low-risk and 224 high-risk samples were compared. The size of the boxes indicates the interquartile range (IQR) which spans from the first quartile (Q1) to the thirst quartile (Q3). The whiskers indicate the range from Q1 + 1.5 x IQR to Q3 –1.5 x IQR and the line is the median. A two-sided unpaired two-sample Wilcoxon test was performed between the two groups. **** $p \leq 0.0001$, ** $p \leq 0.01$, ns: not significant.

References

1. Hornburg, M.; Desbois, M.; Lu, S.; Guan, Y.; Lo, A.A.; Kaufman, S.; Elrod, A.; Lotstein, A.; DesRochers, T.M.; Munoz-Rodriguez, J.L.; et al. Single-cell dissection of cellular components and interactions shaping the tumor immune phenotypes in ovarian cancer. *Cancer Cell* **2021**, *39*, 928-944.e6, doi:10.1016/j.ccell.2021.04.004.
2. Izar, B.; Tirosh, I.; Stover, E.H.; Wakiro, I.; Cuoco, M.S.; Alter, I.; Rodman, C.; Leeson, R.; Su, M.J.; Shah, P.; et al. A single-cell landscape of high-grade serous ovarian cancer. *Nat. Med.* **2020**, *26*, 1271–1279, doi:10.1038/s41591-020-0926-0.
